# IL-2 Inducible Kinase ITK is Critical for HIV-1 Infection of Jurkat T-cells

**DOI:** 10.1038/s41598-018-21344-7

**Published:** 2018-02-16

**Authors:** Anika Hain, Melanie Krämer, René M. Linka, Saeideh Nakhaei-Rad, Mohammad Reza Ahmadian, Dieter Häussinger, Arndt Borkhardt, Carsten Münk

**Affiliations:** 1 Clinic for Gastroenterology, Hepatology and Infectiology, Medical Faculty, Heinrich-Heine University Düsseldorf, Düsseldorf, 40225 Germany; 2 Department of Pediatric Oncology, Hematology and Clinical Immunology, Medical Faculty, Heinrich-Heine University Düsseldorf, Düsseldorf, 40225 Germany; 3 Institute of Biochemistry and Molecular Biology II, Medical Faculty, Heinrich-Heine University Düsseldorf, Düsseldorf, 40223 Germany

## Abstract

Successful replication of Human immunodeficiency virus (HIV)-1 depends on the expression of various cellular host factors, such as the interleukin-2 inducible T-cell kinase (ITK), a member of the protein family of TEC-tyrosine kinases. ITK is selectively expressed in T-cells and coordinates signaling pathways downstream of the T-cell receptor and chemokine receptors, including PLC-1 activation, Ca^2+^-release, transcription factor mobilization, and actin rearrangements. The exact role of ITK during HIV-1 infection is still unknown. We analyzed the function of ITK during HIV-1 replication and showed that attachment, fusion of virions with the cell membrane and entry into Jurkat T-cells was inhibited when ITK was knocked down. In contrast, reverse transcription and provirus expression were not affected by ITK deficiency. Inhibited ITK expression did not affect the CXCR4 receptor on the cell surface, whereas CD4 and LFA-1 integrin levels were slightly enhanced in ITK knockdown cells and heparan sulfate (HS) expression was completely abolished in ITK depleted T-cells. However, neither HS expression nor other attachment factors could explain the impaired HIV-1 binding to ITK-deficient cells, which suggests that a more complex cellular process is influenced by ITK or that not yet discovered molecules contribute to restriction of HIV-1 binding and entry.

## Introduction

Although pharmacological approaches to treating Human immunodeficiency virus (HIV)-1 infection have become increasingly efficient, eradication of the virus in infected individuals remains impossible except in rare circumstances after allogeneic stem-cell transplantation^1^. Antiretroviral therapy does not lead to virus eradication because HIV-1 can establish a highly stable reservoir of latently infected cells^2^. Additionally, highly drug-resistant virus strains are emerging during the treatment of patients, as current treatment approaches target mostly viral proteins only^3^. Thus, the control of HIV-1 by endogenous restriction factors and the dependency on specific host factors have become a focus in modern infectiology^4–6^. Approaches that enhance or reduce such endogenous factors (*e.g*., by influencing natural cellular processes) may be less prone to treatment failure, at least theoretically. Recently, it was shown that inhibition of the interleukin-2 (IL-2) inducible T-cell kinase (ITK) influences HIV-1 infection at early and late stages^7–9^.

ITK is a member of the TEC kinase family, which also includes RLK, BMX, TEC, and Bruton’s tyrosine kinase (BTK)^10^. ITK is selectively expressed only in T-cells and mast cells and is absent in all other human cell types. Because HIV-1 infection requires proper T-cell activation, the need for ITK as a host factor likely is related to its role in integrating signaling pathways downstream of the T-cell receptor and chemokine receptors and regulation of processes important for T-cell activation and differentiation^11,12^. These processes include activation of RHO family GTPases RAC1 and CDC42 and actin polarization^12–15^ and regulation of gene expression^16–18^ and intracellular calcium signaling^19^. Additionally, a lack of up-regulation of integrin adhesion was reported for ITK negative cells, probably because of an inability to recruit LFA-1 integrin and other molecules to the site of T-cell receptor stimulation^20^. Although the immune response in ITK deficient mice is impaired, they can still clear viral infections of vesicular stomatitis virus (VSV) as well as vaccinia and lymphocyte choriomeningitis virus^21^. Indeed, ITK is mainly involved in type 2 helper T-cell (T_H_2) responses, which can mediate asthma and hypersensitivity^22^. Activation of ITK was shown to cause airway hyper-responsiveness and inflammation in mouse models^23^, thus a variety of specific pharmacological ITK inhibitors have been developed and tested in early clinical trials, mostly because of their anti-allergic potential^24–27^.

Our goal was to further explore the relevance of ITK for infection of HIV-1 and identify novel interaction partners that could be explored as targets to interfere with viral replication.

## Results

### ITK knockdown restricts HIV-1 replication in Jurkat T-cells

**Figure 1:**
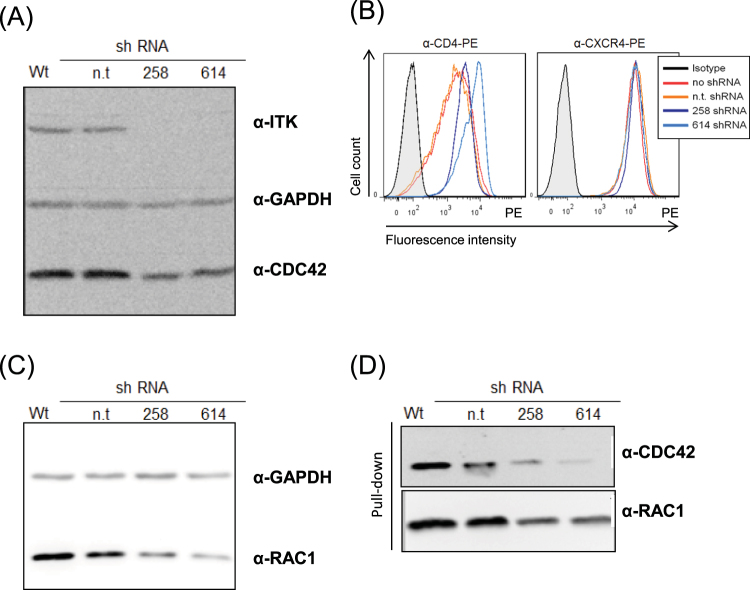
Characterization of ITK knockdown cells. (**A**) Jurkat cells expressing shRNAs targeting ITK or non-target (n.t.) shRNA were assayed for ITK expression *via* immunoblots using an ITK-specific antibody. Immunoblots were co-probed using anti-GAPDH antibody to show equal sample concentrations. (**B**) Expression of HIV-1 receptors CD4 and CXCR4 was analyzed in knockdown and wild-type cells by FACS analysis. Filled histograms represent isotype control and open histograms staining of CD4 or CXCR4 receptor. (**C**) Amount of total CDC42 and RAC1 was assayed by immunoblots using CDC42 and RAC1 specific antibodies. Immunoblots were co-probed using anti-GAPDH antibody to show equal sample concentrations. (**D**) Amount of activated form of CDC42 and RAC1 was assayed by pull-downs (input proteins shown in Fig. 1A and C), followed by immunoblot detection using CDC42 and RAC1 specific antibodies. Full-length blots are presented in Supplementary Fig. 1. Repetition of experiments: for Fig. 1A,C,D five times; for Fig. 1B three times.

To investigate the function of ITK in HIV-1 replication, Jurkat T-cells were stably transduced with lentiviral particles expressing different small hairpin RNAs (shRNAs) targeting ITK. After screening nine different shRNAs (data not shown), we identified two constructs, shITK 258 and shITK 614, that reduced the cellular abundance of endogenous ITK protein (Fig. 1A, Supplementary Fig. 1A). Knockdown of ITK RNA was further confirmed *via* quantitative q-PCR analysis and Ca^2+^ flux measurements (data not shown). The expression of CD4 and CXCR4 on Jurkat cells was determined by flow cytometry. ITK knockdown cells showed regular levels of CXCR4 on the cell surface, but levels of CD4 were moderately higher than in ITK wild-type cells (Fig. 1B). The ability of ITK to regulate RHO GTPases^12^ prompted us to examine the expression and activity of CDC42 and RAC1 in Jurkat ITK knockdown cells. Therefore, we used the RHO binding domain (RBD) of PAK1, known as RAC1/CDC42 effector, to detect the endogenous GTP-bound RAC1 and CDC42. We observed in ITK knockdown cells a down-regulation of RAC1 and CDC42 in total cell lysates (Fig. 1A,C, Supplementary Fig. 1A,B), as well as less GTP-bound protein in pulled down samples (Fig. 1D, Supplementary Fig. 1C,D). Of note, unspecific binding to GST alone was not detectable (data not shown). These data indicate physiological consequences of the loss of ITK protein in RHO GTPase regulation.

**Figure 2:**
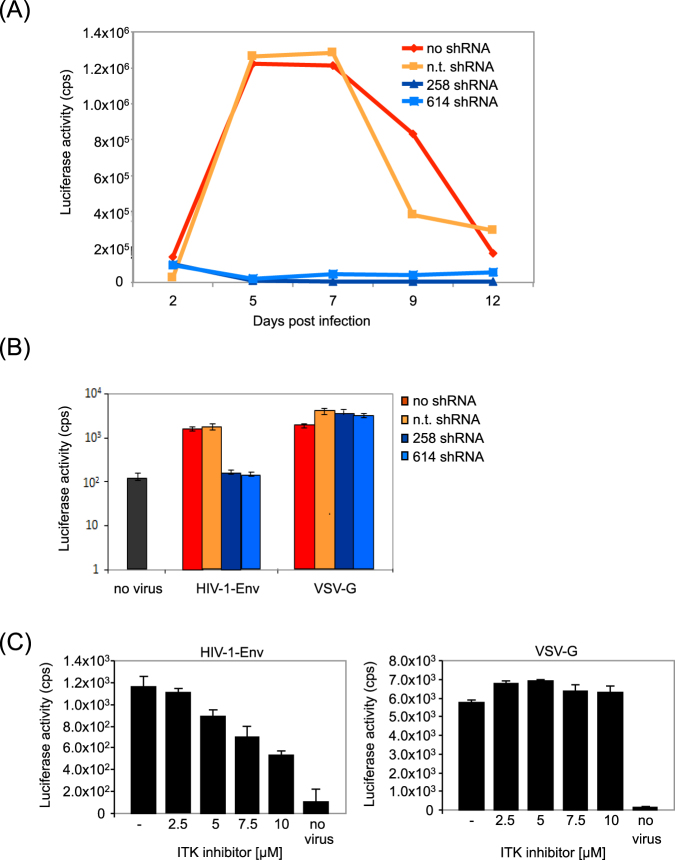
Loss of ITK expression blocks HIV-1 replication in Jurkat cells. (**A**) Replication of HIV-1 in ITK expressing wild-type and non-target (n.t.) shRNA cells was compared with replication in ITK knockdown cells (258/615 shRNA). Cells were infected with an MOI 0.01 and supernatant was collected for 12 days. Virus titer was determined by infecting TZM-bl cells and measuring luciferase activity three days post infection. (**B**) Jurkat cells expressing ITK or no ITK were infected with single-round HIV-1 luciferase-reporter viruses pseudotyped with HIV-1 derived envelope protein (HIV-1-Env) or VSV-G protein. Luciferase activities of infected cells were determined three days post infection. (**C**) Jurkat wild-type cells were incubated with an ITK inhibitor (BIX02524) using different concentrations (0/2.5/5/7.5/10 µM) and transduced with HIV-1 luciferase reporter viruses either pseudotyped with HIV-1 envelope or VSV-G. Luciferase activities of infected cells were determined three days post infection. Repetition of experiments: for Fig. 2A four times; for Fig. 1B three times; for Fig. 2C two times.

To assess HIV-1 replication, cells were infected with replication competent HIV-1 (clone NL4-3) and viral replication was monitored for 12 days. The virus titer was determined by infecting TZM-bl reporter-cells with cell culture supernatant. The results showed that wild-type and non-target (n.t.) shRNA Jurkat cells supported HIV-1 replication, whereas ITK knockdown cell lines were resistant to viral infection (Fig. 2A). In addition, virus replication in these cells were independently monitored by quantification of the reverse transcriptase (RT) activity in the supernatants of infected cells (Supplementary Fig. 2). This experiment confirmed that in ITK knockdown cells the HIV-1 replication is impaired. However, while we detected in cells expressing the shRNA 614 the absence of HIV-1 spread, Jurkat cells with the shRNA 258 showed a delayed and reduced virus production. Together, these results support the notion that ITK regulates multiple steps, early and late, during the HIV-1 infection, as described before^7–9^.

Single-round HIV-1-based luciferase viruses then were used to identify which stage of the viral life cycle was affected by ITK. Infection assays with HIV-1 enveloped particles (from clone L102, a variant of the C-terminally truncated (at amino acid 712) HIV-1 Env strain BH10) showed a strong dependency on ITK expression. HIV-reporter gene activity was highly reduced in the ITK knockdown cells compared to Jurkat cells that expressed ITK. However, transduction of Jurkat cells with VSV-G enveloped HIV reporter viruses resulted in luciferase activity independent of the ITK expression (Fig. 2B). We also tested the ITK inhibitor BIX02524^28^ on HIV-1 infection using the luciferase reporter viruses (Fig. 2C). Jurkat cells treated with the ITK inhibitor showed a concentration-dependent reduced susceptibility for HIV-1 transductions that were mediated by the HIV envelope protein, but infections were not repressed when VSV-G pseudotypes were tested.

These findings indicate that the ITK deficiency caused a specific HIV-1 restriction that involves the viral fusion and entry mechanism and excludes early downstream steps such as reverse transcription, integration, and protein expression.

### Fusion of HIV-1 with the host cell membrane is inhibited in ITK knockdown cells

**Figure 3:**
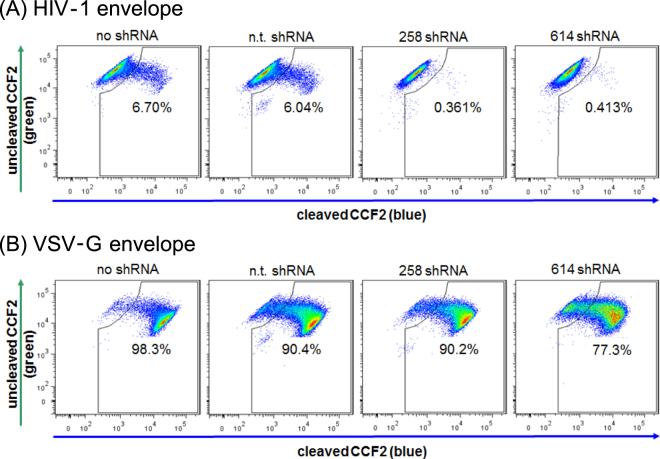
Fusion of HIV-1 enveloped virus with ITK deficient Jurkat cells is inhibited. (**A**) Jurkat cells expressing ITK /no shRNA, (n.t. shRNA) showed HIV fusion rates of 6.04% to 6.7% with HIV-1 envelope pseudotyped ß-lactamase containing viral particles, whereas ITK knockdown cells (258 shRNA, 614 shRNA) showed strong inhibition of viral fusion. (**B**) The same cells were tested for HIV fusion using VSV-G pseudotyped viral particles. Repetition of experiments: Fig. 3 four times.

Because our experiments suggested an ITK-mediated defect in viral entry, virion-based fusion assays were performed as described previously^29,30^. Viral particles were generated with incorporated ß-lactamase-Vpr chimeric proteins. After successful fusion with the host cell membrane, ß-lactamase protein was delivered to the cytoplasm where it cleaved a fluorescence substrate (CCF2). Cleavage of the dye caused a change in the emission spectrum from green (520 nm) to blue (447 nm). The viral fusion rate was calculated by flow cytometry based on the green to blue ratio of the cell population. For wild-type Jurkat and non-target shRNA expressing cells, the fusion rates were 6.7% and 6.04% respectively, when HIV-1 particles with HIV envelope were applied. However, fusion rates of the same virus stock were only 0.38–0.41% with the ITK deficient cells (Fig. 3A). VSV-G pseudotyped viruses that were used as the positive control showed equally high rates of viral fusion for wild-type, non-target shRNA expressing cells and ITK-deficient cells (Fig. 3B).

### Attachment of viral particles to ITK knockdown cells is impaired in a gp120 independent manner

**Figure 4:**
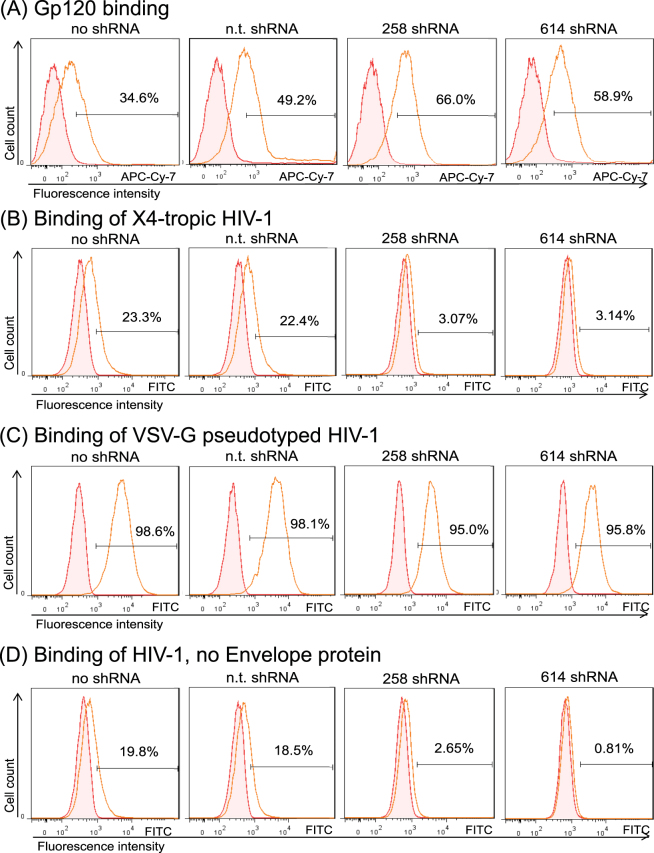
Binding of viral particles but not of gp120 envelope protein is impaired in ITK knockdown cells. Jurkat cells were incubated either with biotinylated gp120 protein (**A**) or GFP labeled viral particles with HIV-1 envelope (**B)**, VSV-G envelope (**C**) or viral particles that were generated without any viral envelope protein (**D**). Data were obtained by FACS analysis of particle or protein incubated cells. Filled histograms represent negative controls composed of cells incubated without gp120 or virus. All figures show one representative experiment out of three repetitions.

Because ITK expression influenced the HIV-1 fusion process, we analyzed the interaction of the gp120 envelope protein of HIV-1 with the host cells. Biotinylated gp120 protein was incubated with ITK expressing and ITK knockdown Jurkat cells. After 1 h of incubation, unbound protein was removed and cell-associated gp120 was visualized by staining with APC-Cy-7-coupled avidin and detected by flow cytometry. Compared to the negative control (cells incubated with biotinylated protein lysate without gp120), approximately 50% (34–66%) of the cells were positive for APC staining. However, no difference in gp120 binding was detected between ITK expressing and non-expressing cells (Fig. 4A).

We then analyzed the attachment of viral particles to target cells. Fluorescent viral particles were produced using viral vectors expressing a GFP-fused Gag polyprotein^31^. These GFP protein containing viruses were incubated with wild-type and ITK deficient cells. After removing unbound virus, cells were fixed with paraformaldehyde and fluorescence was analyzed by flow cytometry. Green HIV-1 particles with HIV-derived envelope protein were detected on the cell surface of ITK expressing wild-type and non-target shRNA cells, as demonstrated by a 22% and 23% increase in GFP fluorescence, respectively. In contrast, viral association with ITK knockdown cells was reduced, and the virus-associated fluorescence of ITK knockdown cells only reached 3% (Fig. 4B). Replacing the HIV-1 glycoprotein with the VSV-G protein resulted in green virions that strongly bound to all Jurkat cells irrespective of their ITK expression (Fig. 4C). Finally, we tested green virus particles that were produced without any viral glycoprotein. These GFP particles weakly bound to ITK knockdown cells, producing a fluorescence of 0.8% and 2.6%, whereas the ITK expressing cells associated strongly with these virions, showing a fluorescence of 19.8% and 18.5% (Fig. 4D).

### Cell surface expression of heparan sulfate (HS) is reduced in ITK deficient cells

**Figure 5:**
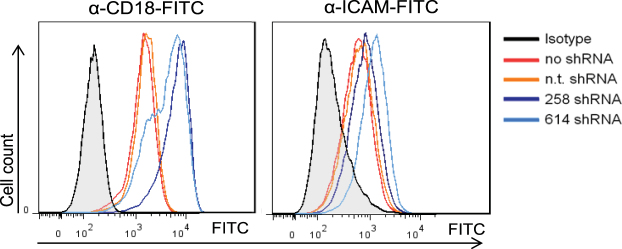
Cell surface expression of LFA-1 and ICAM-1 on Jurkat cells. FACS analysis of expression of CD18 (beta subunit of LFA-1) and ICAM-1 on Jurkat cells expressing ITK (no shRNA, n.t. shRNA) or not expressing ITK (258 shRNA, 614 shRNA) by using anti-CD18-FITC antibody and anti-ICAM-FITC. Filled histograms represent the isotype control. All figures show one representative experiment out of three repetitions.

Our data indicated that ITK knockdown cells have a very early block to HIV-1 infection due to processes affecting viral binding and membrane fusion. Because the expression of HIV receptors CD4 and CXCR4 were comparable in wild-type and ITK knockdown cells, other attachment factors may be affected by ITK. A potential alternative attachment protein is Siglec-1 (CD169)^32^. However, using flow cytometry analysis we found that neither wild-type nor ITK knockdown Jurkat cells expressed Siglec-1 on the cell surface, although it was detected on lipopolysaccharide treated monocyte-derived dendritic cells (data not shown). The presence of lymphocyte function-associated antigen 1 (LFA-1) can increase HIV-1 infection by improving viral attachment^33,34^, and ITK was shown to be involved in regulation of integrins such as LFA-1^20,35^. Therefore, cell surface expression of LFA-1 was analyzed using an anti-CD18 antibody. We also quantified the main interaction partner of LFA-1, ICAM-1, which was reported to enhance the infectivity of HIV-1 by incorporation into viral particles^36,37^. Although the amount of ICAM-1 was only slightly higher, LFA-1 expression was significantly enhanced in ITK knockdown cells compared to wild-type cells (Fig. 5). To test whether LFA-1 is important for HIV-1 infections, Jurkat cells lacking LFA-1 expression were generated using CRISPR-Cas9^38^ (Supplementary Fig. 3A). Single-round reporter virus infections in wild-type and LFA-1 knock-out cells showed that the LFA-1 expression did not change the susceptibility to HIV-1 (Supplementary Fig. 3B). Similar, LFA-1 expression did not modulate the binding of HIV-1 particles to Jurkat cells (Supplementary Fig. 3C). However, by analyzing the spreading replication in both Jurkat cell lines during 18 days using the TZM-bl reporter cells as read-out, LFA-1 wild-type cells showed a higher virus replication compared to the LFA-1 knock-out cells (Supplementary Fig. 3D). LFA-1 deficiency did not impact the cell proliferation as measured with Carboxyfluorescein succinimidyl ester dye (CFSE) fluorescence for 96 hours by flow cytometry (Supplementary Fig. 3E). Thus, we conclude, that absence of ITK may have caused up-regulation of surface LFA-1, which is, however, unlikely to restrict entry of HIV-1.

**Figure 6:**
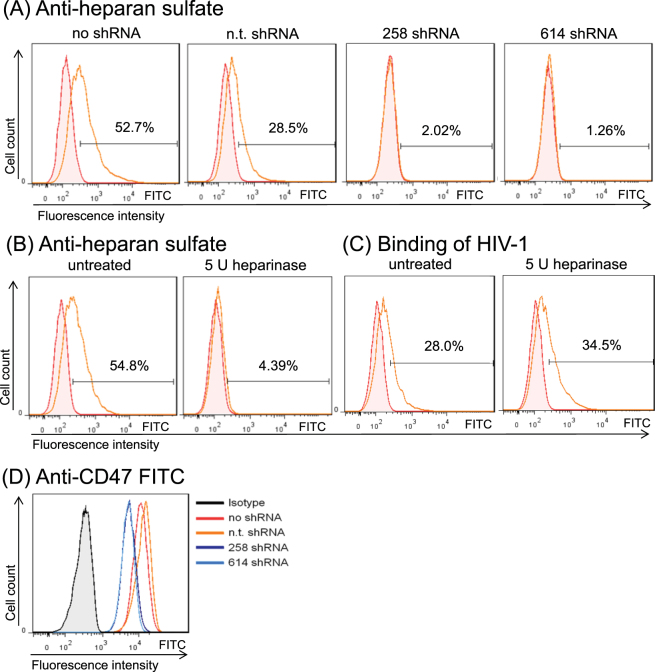
Heparan sulfate expression is missing on ITK deficient cells but is not responsible for reduced viral binding. (**A**) Jurkat cells expressing ITK (no shRNA, n.t. shRNA) or not expressing ITK (258 shRNA, 614 shRNA) were examined by FACS analysis for heparan sulfate on the cell surface using anti heparin sulfate-FITC. (**B**) Jurkat wild-type cells were treated with a mixture of 5 units (U) heparinase I and III and analyzed by FACS again for heparan sulfate expression using anti heparin sulfate-FITC, proofing the efficiency of enzymatic digestion. Filled histograms represent the isotype control. (**C**) Jurkat wild-type cells with enzymatically removed heparan sulfate (5 U heparinase I and III mixture) tested for binding of GFP-labelled HIV-1 virus particles pseudotyped with HIV-1 envelope protein. (**D**) Jurkat cells expressing ITK (no shRNA, n.t. shRNA) or not expressing ITK (258 shRNA, 614 shRNA) were analyzed by FACS analysis for CD47 using anti-CD47-FITC antibody. Filled histograms represent the isotype control. All figures show one representative experiment out of three repetitions.

We also analyzed the presence of HS, a linear sulfated glycosaminoglycan that has been implicated being important for attachment of HIV-1 (for review^39^). HS usually is part of a proteoglycan (HSPG), consisting of a cell surface protein with one or more HS chains covalently attached^40^. Only a small number of HSPGs (<20) have been identified, whereas hundreds of proteins have the capacity to interact with HS (for review^41^). We measured the HS level on the cell surface of our Jurkat cells with and without ITK expression by flow cytometry using an antibody reacting with the 10E epitope. That epitope is present on many types of HS including N-acetylated and N-sulfated glucosamine residues. Much to our surprise, ITK deficient cells lacked most of the HS that was detected on ITK expressing cells. While wild-type cells showed moderate expression, HS was not detectable on knockdown cells (Fig. 6A). To investigate the importance of HS for HIV-1 attachment, HS was enzymatically removed by a mixture of heparinase I and III. Jurkat wild-type cells, which positively stained for HS expression, had a significantly reduced amount of HS molecules on their cell surface after the enzymatic treatment (Fig. 6B). However, the susceptibility to HIV-1 attachment of HS-stripped cells did not differ from that of not digested cells (Fig. 6C). To understand if the lack of HS in the ITK deficient cells affected also the presence of HSPGs, we tested CD47 expression. CD47 is one of the highly expressed proteins in Jurkat cells that is modified by HS^42^. We detected CD47 on all four types of Jurkat cells, with moderately reduced surface levels on ITK knockdown cells (Fig. 6D). Together with previous findings that CD47 depletion did not reduce HIV-1 infections^43^, we conclude that the light reduction of cell surface CD47 expression is unlikely causing the replication block we detected in ITK deficient cells.

## Discussion

The T-cell specific TEC kinase ITK is involved in regulation of various processes, including actin reorganization, Ca^2+^ mobilization, and control of transcription factors. Therefore, ITK plays an important role during cell differentiation and activation (for review see^44^). Because proper T-cell activation is required for HIV-1 infection, a function for ITK as a host factor for HIV-1 replication was expected, and recent studies confirmed a block of HIV-1 at various steps of the viral life cycle after ITK inhibition^7–9^. Here we show that down-regulation of ITK expression *via* RNA interference (RNAi) led to severely reduced HIV-1 expression and spreading in human Jurkat T-cells as well as reduced membrane fusion of HIV-1 caused by an impaired virus particle attachment. Consistent with Readinger *et al*.^7^, our experiments confirm that ITK affects viral entry, but not reverse transcription.

The lower capacity for virus binding might be explained by defects in actin reorganization and receptor clustering, as ITK was shown to be important for control of the actin cytoskeleton and local enrichment of adapter molecules as well as gp120-induced cytoskeleton reorganization^7,22^. In our experiments the typical F-actin depolymerization in Jurkat cells incubated with HIV-1 was detectable in wild-type cells but not in ITK knockdown cells (data not shown). This observation is in agreement with the reduced expression of active RHO GTPases in ITK knockdown cells (Fig. 1C), but the absence of attached viral particles likely contributed to the missing actin depolymerization. In contrast, HIV-1 particles that were pseudotyped with VSV-G were able to undergo membrane fusion and infection in ITK-deficient cells. These observations indicate that the step of viral attachment and fusion is the impaired function in ITK knockdown cells. Although VSV entry also requires actin reorganization mediated by RHO GTPase signaling, the route of entry and the involved molecules differ. HIV-1 enters cells in a pH independent way at the plasma membrane; whereas VSV-G mediates pH dependent fusion *via* endocytosis in clathrin coated vesicles^45^. Thus, for HIV-1 entry the RHO GTPases RAC1, CDC42 and RHOA play a dominant role^6,46–48^, whereas RHOB and RHOC seem to be more important for VSV entry^49^. Our data indicate further that ITK-deficiency in Jurkat cells diminishes a late stage in HIV replication affecting the viral infectivity. In our study, we did not explore this late stage block in detail because we focused on HIV attachment and entry. However, other studies found that after HIV-1 infection ITK deficiency induces a late stage block of replication including virion assembly and release^7,9^. In addition, it was shown that the HIV-1 protein Nef binds and activates ITK, suggesting that Nef may play a role in the recruitment and activation of ITK, and thus contributes to viral egress^8^.

The expression levels of the HIV-1 receptors CD4 and CXCR4 in wild-type and ITK knockdown cells were similar, with slightly higher CD4 levels in the ITK deficient cells. We conclude that the attachment of HIV is not much affected by gp120 binding to CD4/CXCR4, which appears to be needed only to induce membrane fusion. By analyzing alternative attachment factors on Jurkat cells, we found that ITK knockdown cells expressed higher levels of the integrin LFA-1, slightly reduced levels of CD47, and no HS compared to wild-type cells. The membrane glycoprotein LFA-1, an integrin which is composed of the integrin alpha L chain ITGAL (CD11) and the beta 2 chain ITGB2 (CD18) is expressed on T-cells, were it functions in recruitment of the cells to the site of infection and interacts with antigen-presenting cells^50^. For binding to one of the interaction partners ICAM-1 (CD54), ICAM-2 (CD102) or ICAM-3 (CD50), LFA-1 needs to be activated first. During production of HIV-1 cellular ICAM-1 is selectively packaged into new particles by interaction with the viral matrix protein^36^. In addition, elevated attachment and entry of “cell-free” HIV-1 to CD4 T-cells is mediated by incorporated ICAM-1^37,51–54^. Since LFA-1 and ICAM-1 are also integral components of the HIV-1 virological synapse^33,55^, a supramolecular structure which mediates efficient viral transmission between infected and uninfected T-cells^33^, it is speculated that interactions of this adhesion molecules are not only important for infection with free virus particles but also for viral cell to cell transmission. We saw a moderate effect of the LFA-1 deficiency on spreading replication of HIV-1, consistent with the previous observation that T-cells deficient in LFA-1 were less able to support cell-cell transfer of HIV-1^33^. However, our experiments suggest that LFA-1 expression in Jurkat cells is not important for HIV-1 binding and entry. CD47 (also known as integrin-associated protein) is a ubiquitously expressed glycoprotein of the immunoglobulin superfamily that plays a critical role in self-recognition. CD47 was not investigated further in this study, because we found the down-regulation rather moderate on ITK-deficient cells and a previous study demonstrated that CD47-deficiency does not affect HIV-1 replication^43^.

The anti-HS antibody used in our study recognized the 10E4 epitope that is present in many types of HS. Thus, it is likely that ITK knockdown cells do not express HS on the cell surface. Alternatively, ITK knockdown cells could express HS variants that do not react with the anti-HS antibody. HS moieties on cell surfaces are known to provide attachment sites for many viruses, including foamy retroviruses^56,57^. In line with the importance of HS for foamy virus infection, infection of ITK knockdown cells by prototype foamy viruses revealed a 10-fold restriction compared to infection of ITK-expressing Jurkat cells (data not shown). Studies have also shown that HS is important for attachment of HIV-1 to target cells^39,58–63^. Most of these studies concluded that HS interacts with the viral envelope protein; however, other reports show that attachment of envelope protein-free HIV particles to cells is caused at least in part by HS^64,65^. In these studies, treatment of HeLa cells with heparinase I reduced binding of envelope protein free HIV particles by 25%, and the highest heparinase concentrations reduced binding by 50%^64^. We found that HIV-1 particles without a viral glycoprotein bound to Jurkat wild-type cells but not to ITK knockdown cells and that the enzymatic removal of HS from the cell surface of Jurkat cells by a mixture of heparinase I and III did not reduce this binding. These data indicate that Jurkat T-cells contain an HS-independent attachment factor that does interact with the viral membrane. This factor, however, has yet to be identified. The striking correlation between very low HS expression in ITK knockdown cells and the absence of attached HIV-1 particles may point to a more complex mechanism that cannot be completely described by cell surface staining using the anti-HS antibody. Finally, binding of the HIV particles in Jurkat T-cells might be regulated by a HS-modified protein, and the observed lack of HS may be the result of down-regulation of such a protein.

It appears that this unknown factor mediates HIV-1 attachment in a gp120 Env-independent way. We hypothesize that HIV with viral glycoproteins that weakly bind to receptors, *e.g*., HIV-1 Env BH10, need this additional attachment for infection of Jurkat cells and infection of ITK-deficient Jurkat cells may need viral glycoproteins that have a stronger interaction with receptors, e.g., VSV-G. ITK is not expressed in HEK293T or HeLa cells; however, if these cells are genetically modified to express CD4 and CXCR4, they are fully permissive for HIV-1 infection (data not shown). In addition, we found ITK expression in other HIV-1 permissive T-cell lines to be absent or much lower than in Jurkat cells (data not shown). These observations suggest that ITK is not strictly required for HIV-1 infection and that the ITK-dependence of HIV-1 may reflect an entry-associated pathway that is present in Jurkat cells and maybe different in other cell lines. Altogether, our results support the premise that ITK is an important protein that modulates the permissivity of Jurkat T-cells for HIV-1 infection that involves an unknown mechanism for HIV-1 attachment.

## Methods

### Cell culture

Human embryonic kidney cells (HEK293T, ACC 635; DSMZ, German Collection of Microorganisms and Cell Cultures, Braunschweig, Germany) and TZM-bl cells (26) were cultured in DMEM (Biochrom, Berlin, Germany) containing 10% FCS, 100 units/ml penicillin, 100 µg/ml streptomycin and 2 mM L-glutamine. Jurkat clone E6-1 (TIB-152, American Type Culture Collection, Manassas, USA) was cultured in RPMI medium 1640 containing 2 mM Glutamax (Thermo Fisher Scientific, Darmstadt, Germany) supplemented with 10% FCS, 100 units/ml penicillin, and 100 µg/ml streptomycin. For digestion of cell surface heparan sulfates 1,5 × 10^5^ cells were incubated with 5 U heparinase I and III from *Flavobacterium heparinum* (H3917, Sigma-Aldrich, Taufkirchen, Germany) for 1 h at 37 °C

### Transfections and production of viral particles

Transfections were performed in 6-well plates seeded with 5 × 10^5^ cells/well using Lipofectamine LTX (Life Technologies, Darmstadt, Germany) according to the manufacturer’s instructions. 48 h post transfection cells were used for further experiments. If cells were transfected to produce viral particles, the virus containing supernatants were collected and filtered (0.45 µm pore size). Virus titer was either determined by using the Cavidi HS lenti RT kit (Cavidi Tech, Uppsala, Sweden) or by infection of TZM-bl reporter cells. Concentrated virus supernatant was obtained by centrifugation through a sucrose cushion (20% sucrose in PBS) at 40000 g for 2 h at 4 °C.

### Small hairpin RNA

Small hairpin RNA (shRNA)-mediated ITK knockdown was carried out using a set of lentivirus particles expressing different shRNAs against ITK (MISSION shRNA cat. no. SHGLY-NM_005546; Sigma), or non-target shRNAs (cat. no. SHC002; Sigma). Lentivirus particles were produced by transfecting HEK293T cells with 700 ng shRNA expression plasmid, 280 ng of pMDLg/pRRE^66^ packaging construct, 110 ng pRSV-Rev^66^ and 60 ng of VSV-G expression plasmid. Jurkat cells were spin-transduced *via* centrifugation for 1 h at 31 °C at 2000 rpm by lentivirus particles expressing shRNA. Two days after transduction cells were cultured in the presence of 2 μg/ml puromycin (Applichem, Darmstadt, Germany) for a two-week selection period.

### Calcium measurements

Calcium flux analyses of human T-cells were performed according to^67^. Briefly, 3 × 10^5^ cells/mL in phenol red-free RPMI 1640 (Life Technologies) containing 10% FCS were loaded with 5 µg/mL Indo-1 AM (MoBiTec, Göttingen, Germany) at 37 °C for 45 min, followed by an additional incubation for 45 min in medium without Indo-1. Cells were kept on ice before equilibration at 37 °C for 5 min, directly before measurement. Changes in intracellular calcium were monitored using a flow cytometer LSRI (BD Biosciences, Heidelberg, Germany). Cells were illuminated using the 325 nm laser-line of a helium-cadmium laser. Fluorescence emissions at 405/30 nm (calcium-bound Indo) and 510/20 nm (free Indo) were detected simultaneously, analyzing the ratio of bound to free Indo over time. After monitoring the baseline activity for 1 min, the cells were stimulated by 10 µg/mL CD3 mAb (clone UCHT1, BD Biosciences) and cells were measured for another 6 min. To confirm proper Indo-1 loading, the cells were then treated with 10 µg/mL ionomycin (Sigma-Aldrich). Kinetics were analyzed using FlowJo v7.6.3 software (Tree Star, Ashland, USA).

### q-PCR

Expression levels of ITK transcripts in Jurkat cells stably expressing sh ITK RNA were quantified by q-PCR using the Applied Biosystems 7500 Real-Time PCR system (Applied Biosystems, California, United States). Total RNA was isolated from cells *via* the RNeasy Mini kit (Qiagen, Hilden, Germany) and 1 µg of total RNA was used to produce cDNA by Quantitect Reverse Transcription Kit (Qiagen). Q-PCR-reactions with cDNA were performed in triplicates using the SYBR green master mix (Applied Biosystems) according to the manufacturer’s instructions. ITK was amplified with the primers ITK_Exon1_F1 (5′-TGAACAACTTTATCCTCCTGGAAGA) and ITK_Exon1_R2 (5′-GGTTAACACAAAGAAGCGGACTTTA). After initial incubations at 50 °C for 2 min and 95 °C for 10 min, 40 cycles of amplification were carried out for 15 s at 95 °C, followed by 1 min at 60 °C. ITK levels were normalized to the human reference gene hypoxanthine-guanine phosphoribosyltransferase1 (primers HPRT1_fw 5′-GCTTTCCTTGGTCAGGCAGT and HPRT1_rv 5′GCTTGCGACCTTGACCATCT).

### GST pull-down

PAK1-RHO binding domain (RBD) was expressed in *E. coli* as fused GST protein in the pGEX-2TK plasmid (GE Healthcare, Munich, Germany). The total bacterial lysate was prepared using standard protocols and the quality was checked in SDS-Gels^68,69^. Bacterial lysates containing the PAK1-RBD were used as a bait and subsequently to pull-down activated GTP-bound CDC42 or RAC1 from total Jurkat cell lysates (pray) including the wild-type, scramble and ITK knockdown cells^70^.

### Immunoblot analyses

Cells were lysed in radioimmunoprecipitation assay (RIPA) buffer (25 mM Tris-HCL [pH 7.6], 150 mM NaCl, 1% NP40, 1% sodium deoxycholate [SDS], plus protease inhibitor cocktail set III [Calbiochem, Darmstadt, Germany]). Protein concentration of the lysates was quantified by using Bradford reagent (Applichem). Samples were separated by SDS-Page and transferred to polyvinylidene difluoride (PVDF) membranes. Membranes were probed with rabbit anti-ITK (ab32113, Abcam, Cambridge, United Kingdom, 1:2000), goat anti-GAPDH (EB06377, Everest Biotech, 1:20,000), rabbit anti-CDC42 (#24645, Cell Signaling, Cambridge, United Kingdom, 1:500) or mouse anti-RAC1 (88751, BD Biosciences, 1:500) followed by horseradish peroxidase-conjugated rabbit anti-mouse antibody or goat anti-rabbit antibody (GE Healthcare) or rabbit anti-goat antibody (R131HRP, Acris), and developed with ECL chemiluminescence reagents (GE Healthcare).

### Viral replication

Replication competent virus stocks were generated by transfecting HEK293T cells with 2 µg pNL4-3^71^. Jurkat T-cells were infected with MOI 0.01 or 0.1 and kinetics of viral spreading was analyzed for 14–18 days. Virus titer was either determined by using the Cavidi HS lenti RT kit (Cavidi Tech, Uppsala, Sweden) or by infection of TZM-bl reporter cells.

### Luciferase assay

To generate luciferase-reporter-virus 500 ng pMDLg/pRRE packaging plasmid together with 250 ng pRSV-Rev^66^ and luciferase encoding transfer-vector pSIN luc IRES GFP were co-transfected in 293 T cells. For envelope expression either 300 ng VSV-G expression plasmid (pMD.G^72^) or plasmid encoding L102, a variant of the C-terminally truncated (at amino acid 712) HIV-1 Env strain BH10 (pcL102^73^) was co-transfected. Two days after transfection viral supernatant was harvest and used to infect Jurkat cells. Luciferase activity of Jurkat cells was assayed three days post transduction using the *Steady-Glo Luciferase Assay System* (Promega) according to the manufacturer’s instructions in a MicroLumat Plus luminometer (Berthold Detection Systems, Pforzheim, Germany). Transductions were performed in triplicates; the means and standard deviations of each triplicate are shown. ITK inhibitor BIX02524 (compound 6 in^28^) was tested on HIV-1 infection in Jurkat cells using the luciferase reporter viruses either pseudotyped with HIV-1 envelope or VSV-G. 50 µl cells were preincubated with 1 µl BIX02524 in DMSO (0/5/10/15/20 µM) for 45 min, after that 50 µl of media with virus was added. Infection was performed for 6 h, and then cells were washed three-times and further cultured for 3 days.

### Cell proliferation

Cells were loaded with cell-tracer violet fluorescence dye CFSE (CellTrace CFSE, Thermo Scientific, Schwerte, Germany) following manufacturer’s recommendations, and decrease of fluorescence was monitored by flow cytometry with a 488-nm excitation source (BD FACS canto II, BD Biosciences, analysis were done using the software FlowJo 7.6.3 (Tree Star, Ashland, USA)). Cells were grown under normal culture conditions and fluorescence of CFSE was measured in a time interval of 24 hours, beginning directly after staining and ending after 96 hours. As negative control, cells were incubated 1 h before CFSE staining with 100 µg/ml Cisplatin (Accord Healthcare, Freilassing, Germany), a cytostatic drug which inhibits cell growth.

### CRISPR/Cas9

For LFA-1 knock-out, Cas9 and sgRNA (TGCCCGACTGGCACTGATAGAGG) targeting ITGAL (pLenti-ITGAL) were delivered into Jurkat cells by lentiCRISPRv2-based lentiviral transduction^38^. Lentiviral particles were produced by transfecting HEK293T cells with 800 ng pLenti-ITGAL, 800 ng packaging construct psPAX2 (Trono laboratory, obtained from Addgene, Cambridge, USA) and 300 ng VSV-G expression plasmid. Jurkat cells were spin-transduced *via* centrifugation for 90 min at 1200 rpm at room temperature. Three days after transduction medium was replaced with medium containing 2 μg/ml puromycin (Applichem, Darmstadt, Germany).

### ß-lactamase-based virion fusion assays

ß-lactamase containing viral particles where generated by transfecting 293 T cells with 3rd generation lentiviral plasmids; pMDLg/pRRE and pRSV-Rev for packaging, pSIN PPT CMV Luc ires GFP as viral genome, pMD.G or pL102 for pseudotyping and pMM310 for ß-lactamase-Vpr chimeric protein expression^29^. Two days post transfection viral supernatant was removed and concentrated 10 fold by centrifugation through a 20% sucrose gradient. For fusion assays 1 × 10^6^ Jurkat cells were seeded in a 24 well plate, in a total volume of 500 µl. Then 150 µl of HIV-enveloped or 80 µl VSV-G pseudotyped virus was added. After incubation for 3.5 h at 37 °C cells were washed twice with CO_2_ independent, serum-free media. To allow uptake of the fluorescence substrate, cells were resuspended in 500 µl loading solution, which was prepared directly before use and consists of: CO_2_ independent, serum-free media, 10 mM HEPES, 1% probenecid, 0.015% solution A (CCF2-AM) and 0.08% solution B (100 mg/ml Pluronic-F127R, 0.1% acetic acid). Solution A and B were obtained from the *GeneBLAzer Detection* Kit from Invitrogen. After incubation for 15 h at 25 °C, cells were washed with PBS and fluorescence was measured with BD FACS canto II (BD Biosciences) analysis were done using the software FlowJo 7.6.3 (Tree Star, Ashland, USA).

### Biotinylation

HIV-1 envelope protein was obtained from the NIH AIDS Reagent Program (HIV-1 gp120IIIB (CHO), No. 11784) or purified with Ni-NTA Agarose beads from cell lysate after transfection with pEFgp120-linker-myc-6HIS plasmid. Proteins were labeled with activated NHS-biotins (EZ-link Sulfo-NHS-Biotion, 21326, Thermo Fisher Scientific, Waltham, United States), which covalently binds to primary amines. 5 µg protein and 100-fold molecular excess of biotin were incubated 30 min rotating at room temperature, following manufacturer’s recommendations. Unbound biotin was removed by washing using desalting columns (UFC503024, Merck Millipore, Darmstadt, Germany). Lysate of un-transfected 293 T cells containing no gp120 protein was processed in parallel as negative control.

### gp120 binding assay

To analyze attachment of biotinylated gp120 protein to Jurkat cells, 5 × 10^5^ cells were washed and incubated with 5 µg protein at RT for 1 h in a total volume of 100 µl. After washing cells were re-suspended in 150 µl buffer containing 2 µg streptavidin APC-C7 (554063, BD Bioscience) and incubated for 30 min at RT to stain attached gp120. After repeated washing 1% paraformaldehyd was used for fixation overnight and fluorescence was analyzed by flow cytometry. For binding and washing HEPES^++^ buffer was used (binding buffer: 50 mM HEPES, 5 mM MgCl_2_, 1 mM CaCl_2_, 5% BSA, 0.1% NaN_3_, washing buffer: 50 mM HEPES, 5 mM MgCl_2_, 1 mM CaCl_2_, 5 mM NaCl).

### Attachment assay

To study binding of viral particles to Jurkat cells, virus with Gag-EGFP fusion protein was generated by transfection of 293 T cells with 800 ng pCHIV.eGFP(Env−) and 800 ng pCHIV(Env−)^31^ and co-expression of expression plasmids for HIV Env L102 or VSV-G. Fluorescent virus was incubated with target cells for 2 h at 37 °C. Cells were washed two times with PBS and re-suspended in PBS 2% PFA for flow cytometry analysis.

### Expression of cell surface molecules

Amount of cell surface molecules was quantified by immunofluorescence staining and FACS analysis. Therefore, 5 × 10^5^ Jurkat cells were washed once and re-suspended in 100 µl PBS. For CD4 staining 10 µl mouse IgG anti-hCD4PE (Clone MT310, Dako) was added and cells were incubated 30 min at 4 °C. For CXCR4 staining 10 µl mouse IgG anti-hCXCR4 PE (555974, BD Biosciences) was used and cells were incubated 15 min at 37 °C. LFA-1 was stained with 2 µl FITC-labeled anti-CD18 antibody (Beckman Coulter) and ICAM-1 with 2 µl anti-ICAM-1 (CD54) clone HA58 FITC (eBioscience, San Diego, USA), both for 30 min at room temperature. For labeling of ICAM-1, coupled anti-mouse secondary antibody was used before fixation (Invitrogen). 2 µl anti-human SIGLEC1/CD169 (clone 7-239, eBioscience) and 2 µl anti-CD47-FITC (clone B6H12, eBioscience) were incubated for 45 min at RT. Staining of heparan sulfate (clone F58-10E4, Amsbio, Abingdon, United Kingdom) was performed for 60 min at 4 °C. As Isotype control 10 µl mouse IgG Iso PE, mouse IgG Iso FITC (BD Biosciences) or mouse IgG Iso APC (clone P3.6.2.8.1, eBioscience) was used. After washing twice with PBS, cells were re-suspended in PBS 2% PFA and fluorescence was measured with BD FACS canto II.

### Data Availability

The datasets generated during and/or analysed during the current study are available from the corresponding author on reasonable request.

## Electronic supplementary material

Supplementary Figures
