# Supplementary material for: IL-2 Inducible Kinase ITK is Critical for HIV-1 Infection of Jurkat T-cells
Source: Sci Rep. 2018 Feb 16;8:3217. doi: 10.1038/s41598-018-21344-7 (PMC5816632; doi:10.1038/s41598-018-21344-7)

IL-2 Inducible Kinase ITK is Critical for HIV-1 Infection of Jurkat T-cells

Anika Hain, Melanie Krämer, René M. Linka, Saeideh Nakhaei-Rad, Mohammad Reza Ahmadian, Dieter Häussinger, Arndt Borkhardt, and Carsten Münk

Legends Supplementary Figures

Supplementary Fig. 1: Full-length blots from Fig. 1A, 1C and 1D. (A) Uncropped blot from Fig. 1A. (B) Uncropped blot from Fig. 1C. (C) Uncropped blot from Fig. 1D, top part. (D) Uncropped blot from Fig. 1D, bottom part.

Supplementary Fig. 2: Replication of HIV-1 in Jurkat cells. Spreading replication of HIV-1 in ITK expressing (non-target, n.t., shRNA) cells and ITK-deficient cells (258 shRNA, 614 shRNA). Cells were infected with an MOI of 0.01 and supernatant was collected for 14 days. Virus production was determined by measuring the reverse transcriptase (RT) activity in the culture supernatant. Figures show one representative experiment of the three repetitions.

Supplementary Fig. 3: LFA-1 knock-out cells support HIV-1 infection. (A) Cell surface expression of LFA-1 on wild-type and LFA-1 knock-out (KO) cells. FACS analysis of expression of CD18 (beta subunit of LFA-1) by using a ti-CD18-FITC antibody. Filled histograms represent the isotype control. (B) Jurkat cells expressing LFA-1 or not were infected with single-round HIV-1 luciferase-reporter viruses pseudotyped either with HIV-1-derived envelope protein (HIV-env) or VSV-G protein. Luciferase activity of infected cells were determined three days post infection. Background signal of uninfected cells was subtracted. (C) WT and LFA-1 KO cells were incubated with GFP-labeled HIV-1 particles with HIV-1 envelope. Data were obtained by FACS analysis of particles incubated cells. Filled histograms represent negative controls composed of cells incubated without virus. (D) Replication of HIV-1 in LFA-1 wild-type and KO Jurkat cells. Cells were infected with an MOI of 0.01. Virus titer was determined by infecting TZM-bl cells and measuring luciferase activity. (E) Cell proliferation. Wild type and LFA-1 KO Jurkat cells were loaded with cell-tracer violet fluorescence dye CFSE, and decrease of fluorescence was monitored by flow cytometry. As negative control, cells were incubated Cisplatin, a cytostatic drug which inhibits cell growth. All figures show one representative experiment of the three repetitions.

Supplementary Fig. 1

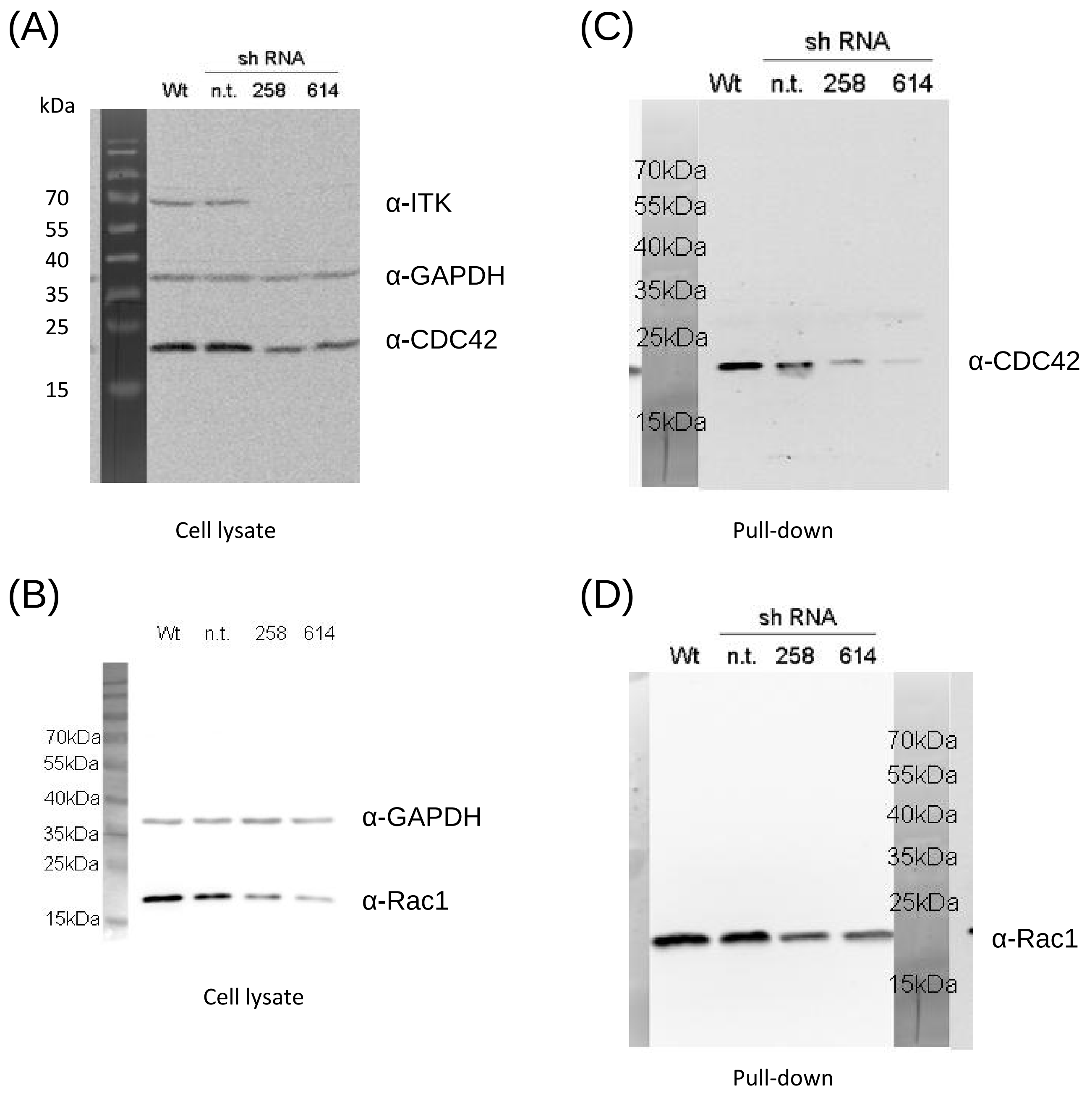

Supplementary Fig. 2

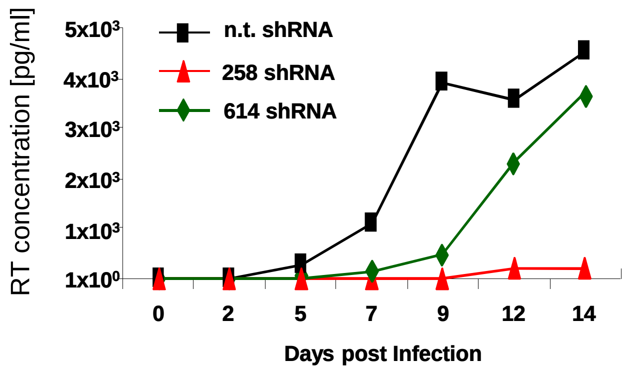

Supplementary Fig. 3

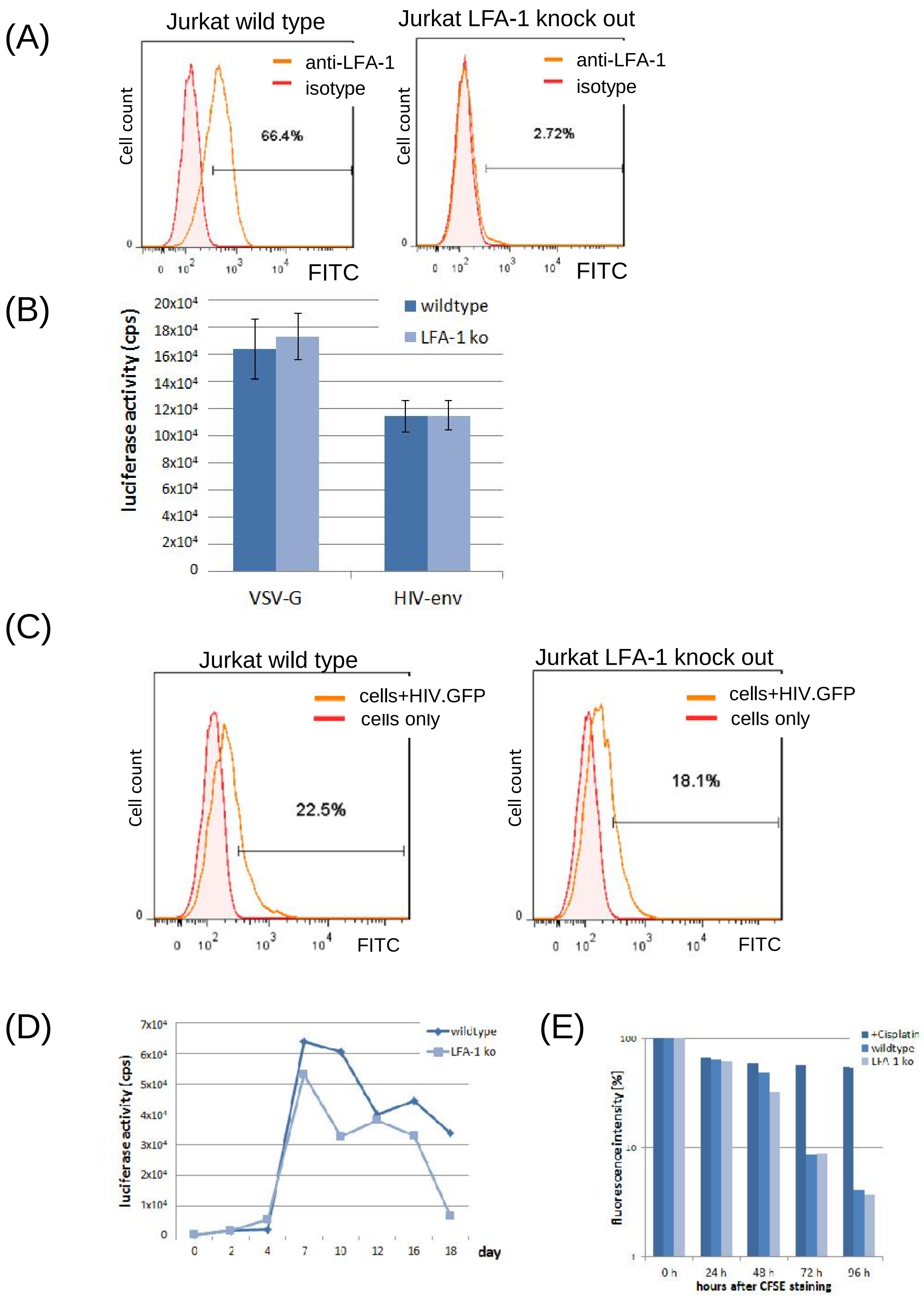

Supplement: Supplementary file 1 — Supplementary Figures [file 41598_2018_21344_MOESM1_ESM.pdf]
